# Knowledge, attitude, and practice of dental health professionals toward dental impression disinfection protocol during the COVID-19 pandemic in Saudi Arabia– a cross-sectional study

**DOI:** 10.1186/s12909-024-05238-z

**Published:** 2024-03-06

**Authors:** Abdullah Salman Binassfour, Mohammad Abdul Baseer, Navin Anand Ingle

**Affiliations:** Preventive Dentistry Department, College of Dentistry, Riyadh Elm University, 11681 Riyadh, Saudi Arabia

**Keywords:** COVID-19, KAP study, Dental impression, Disinfection, DHPs, Coronavirus prevention, Knowledge, Attitude, Practice

## Abstract

**Background:**

Despite the updated guidelines on dental impression disinfection protocols during the COVID-19 pandemic, adherence to such procedures has not been studied among dental health professionals in Saudi Arabia. Understanding DHPs’ knowledge, attitudes, and practices regarding COVID-19 is crucial in assessing a willingness to adhere to the recommendations provided by health authorities in mitigating the spread of COVID-19 via dental impressions impacting patient safety and infection control measures. Hence, this study aimed to assess dental health professionals’ (DHPs) knowledge, attitudes, and practices (KAP) toward dental impression disinfection protocol during the COVID-19 pandemic in Saudi Arabia.

**Methods:**

A cross-sectional study using an online survey was conducted from 14 December 2022 to 21 March 2023 among practicing dentists, dental assistants (DA), dental laboratory technicians (DT), and dental hygienists in Saudi Arabia. A validated and reliable questionnaire that consisted of 38 items along with demographic variables was prepared to collect the data. Using Google Forms, a questionnaire link was prepared and shared on the social media platforms of DHPs in Saudi Arabia. A descriptive analysis was conducted to report the percentages and frequencies. The mean knowledge, attitude, and practice scores were analyzed using an Independent t-test, ANOVA, and Pearson’s correlation tests.

**Results:**

A total of 718 DHPs voluntarily participated in the survey. Most of the DHPs exhibited average knowledge 392 (54.6%), neutral attitudes 393(54.7%), and adequate 549 (76.5%) practice towards dental impression disinfection protocol. The mean knowledge score differed significantly across nationality (*p* = 0.013), type of DHPs (*p* < 0.001), qualification (*p* = 0.045), and experience (*p* = 0.028) of the study participants. Significant differences in attitude towards impression disinfection were observed in different age groups (*p* = 0.002), qualifications (*p* = 0.015), and experiences (*p* = 0.024) of the DHPs. Similarly, practice varied across different age groups (*p* = 0.010), nationality (*p* = 0.013), type of DHPs (*p* = 0.019), qualification (*p* = 0.044), experience (*p* = 0.041), and COVID-19 Infection (*p* = 0.006). Moreover, a significant positive correlation between knowledge-attitude (*r* = 0.258, *p* < 0.01), knowledge-practice (*r* = 0.283, *p* < 0.01), and attitude-practice (*r* = 0.196, *p* < 0.01) was observed.

**Conclusion:**

DHPs considered in this study demonstrated average knowledge and attitudes toward impression disinfection, requiring improvement through continuous dental education and training. However, they displayed acceptable dental impression disinfection practices during the COVID-19 pandemic. It is highly recommended that continuing education programs should mainly reinforce the knowledge of sodium hypochlorite, iodophor, and phenolics and their concentrations to be used as an impression disinfectant. Additionally, it should focus on techniques of disinfecting elastomeric, hydrocolloid, zinc oxide and eugenol, and impression compound materials to mitigate the spread of COVID-19 based on Saudi ministry of health guidelines.

## Background

Dental impression materials are widely used in dentistry. Making impressions is a crucial procedure in dental care, particularly when it pertains to the replication of oral structures. During the impression procedure, impression materials frequently come into contact with saliva and blood, potentially contaminating infectious diseases such as AIDS, herpes, hepatitis, tuberculosis [1], and SARS-CoV-2. Dental impressions that come into contact with a COVID-19-infected patient’s saliva or blood can lead to the contamination of stone casts. This contamination poses a risk of infection to dental personnel who handle or work with these impressions or models. The individuals involved in taking contaminated casts have the potential to cause cross-contamination, resulting in the transfer of pathogens from one patient to another patient’s casts and, ultimately, to the dentist and other patients. Hence, COVID-19 infection control is a crucial and indispensable concern within the dental practice, mitigating the transmission of SARS-COV-2 infections between patients and safeguarding the well-being of dental healthcare providers.

Several chemicals and protocols were used to disinfect different types of impression materials. The impressions are washed correctly in tap water to remove impurities as soon as they are removed from the patient’s mouth. Although cleaning with tap water was found to diminish germs, it did not eradicate the impressions’ infection potential [2, 3]. Therefore, disinfection of dental impressions is required to minimize cross-contamination between patients and dental personnel in dental offices and laboratories. The American Dental Association (ADA) recommends disinfecting dental impressions soon after removing them from the patient’s mouth (ADA 1996).

The literature describes various methods for disinfecting impressions, including chemical disinfection, microwave treatment, autoclaving, and ultraviolet radiation. Each approach has advantages, disadvantages, and effects on impression materials and casts. The two most common chemical disinfection methods are immersion and spraying, which include the use of alcohols, aldehydes, phenols, chlorine, iodide, and ammonium. The immersion disinfection method is effective but not recommended for hydrophilic impression materials like hydrocolloids and polyethers as they can absorb the disinfectant solution, leading to the dimensional inaccuracy of the impression. Another disadvantage of immersion disinfectants is that they should be disposed of after each use; it is time-consuming and expensive. Spray chemical disinfection decreases dimensional changes, notably in hydrocolloid and polyether impression materials. This method utilizes less disinfection solution and may not reach undercuts; therefore, it may not disinfect the impression material effectively. Additionally, chemical disinfectants need fresh preparation and have a low shelf life [4].

The chemical technique is the most commonly employed since it involves spraying or immersing an impression in chemical disinfection. The disinfectants in a variety of concentrations may be used, including glutaraldehyde (0.5%, 2%, 2.2%, and 2.45%), sodium hypochlorite (NaOCl) (0.5%, 0.525%, 1%, 4%, and 5.25%), iodophors (5 and 10%), phenols (7%), chlorine compounds (0.2% chlorhexidine), and hydrogen peroxide (0.5%), etc. It has been found that glutaraldehyde disinfection was shown to remove microorganisms from the surface of alginate efficiently and additional silicone impression materials without affecting their dimensional stability [5].

Steam autoclaving was deemed an appropriate technique, mainly when the impressions were intended for use in the production of removable prostheses. At 134 °C, the standard settings can eradicate most bacteria, spores, viruses, and fungi in three minutes. The disadvantage is that the custom containers used for the impression materials deformed at high temperatures, causing the impression material to change in dimension substantially [6, 7].

Ultraviolet (UV) light has been recognized as a highly recommended modality for disinfecting impressions due to its proven efficacy without compromising the material’s dimensional stability. The efficacy of disinfection is influenced by various factors, including the duration, magnitude, moisture levels, and exposure to ultraviolet (UV) radiation contact from microorganisms [4]. Microwave disinfection is a very efficient and versatile approach characterized by speed, simplicity, and affordability. An inherent drawback of this procedure is that it displayed more significant dimension changes compared to the autoclave method and chemical sterilization [1, 4].

The theoretical framework for the study is based on the KAP (Knowledge, Attitude, and Practice) model established in 1960 to analyze changes in health behavior. According to this model, human behavior comprises three successive processes: acquiring knowledge, generating attitudes, and forming behaviors [8]. Given KAP theory, the spread of COVID-19 would undoubtedly be influenced by dental professionals’ behavioral practices and available knowledge and information essential to the action [9].

Knowledge has been thought to influence behavior. A high dental Impression disinfection knowledge leads to a positive attitude, which leads to better practice compliance with impression disinfection to prevent and control the spread of SARs-COV 2 between patients and DHPs. Therefore, knowledge, attitudes, and practice studies regarding COVID-19 are crucial in identifying the knowledge gap and DHP’s willingness to adopt COVID-19 infection control recommendations provided by health authorities in mitigating the spread via dental impressions of the patients.

Given the documented morbidity and mortality rates among healthcare professionals who have contracted COVID-19, it is reasonable to infer that dental personnel may be at an increased risk of cross-contaminating SARS-CoV-2 infection due to the inherent nature of their profession. Consequently, numerous research studies have advocated the implementation of diverse protocols for effectively disinfecting dental impression materials [10–14].

Numerous disease control organizations (ADA/CDC), including the Ministry of Health of Saudi Arabia, have recommended comprehensive preventive measures in the field of dentistry [15–17] to reduce the risk of COVID-19 infection among dentists. Several studies [18, 19] have previously reported that dentists lack knowledge, attitudes, and perceptions regarding viral infection control including COVID-19 [20, 21]. All DHPs must acquire contemporary knowledge about impression disinfection protocols amidst the ongoing pandemic.

Saudi Arabian ministry of health’s manual of infection prevention and control in dental settings recommends that the dental impressions should be promptly cleaned and disinfected upon removal from the mouth. It is a must to disinfect the impression using the appropriate material and technique, since not all impression materials can be effectively disinfected using the same disinfectant. Thus, it is advised to use disinfectants such as sodium hypochlorite, iodophor, and phenolics in different concentrations and techniques for disinfecting elastomeric, hydrocolloid, Zinc Oxide, and Eugenol (ZOE), and impression compound impression materials [22]. Despite the guidelines on dental impression disinfection protocols during the COVID-19 pandemic, adherence to such guidelines has not been studied among DHPs in Saudi Arabia. It is essential to explore the impression of disinfection awareness and practices among DHPs during this pandemic.

Hence, this study aimed to assess dental health professionals’ knowledge, attitudes, and practices toward dental Impression disinfection protocol during the COVID-19 pandemic in Saudi Arabia.

## Methods

### Ethical approval

The ethical committee of research and innovation center of Riyadh Elm University provided formal approval for the study after thorough review of the research proposal (IRB number: FPGRP/2022/701/807/776). The DHPs participation in the study was voluntary. The purpose of the study, the methods of data acquisition, and the potential risk and benefits were all explained in detail to the DHPs. An implied informed consent to participate in the study was obtained from the DHPs by submitting the completed survey. The identities and responses of DHPs were kept confidential and anonymous by coding the information to safeguard privacy and prevent any unintentional disclosure of the data. Additionally, participants were informed about the use of data for scientific research and publication purposes only. The data was password-coded, securely stored in the principal investigator’s google drive, and not disclosed to unauthorized individuals. This study was conducted as per the Helsinki Declaration.

### Study design

The present study employed a descriptive cross-sectional survey design to assess the knowledge, attitudes, and practices of DHPs regarding dental impression disinfection protocols. This study was carried out in accordance with the Strengthening the Reporting of Observational Studies in Epidemiology (STROBE) guidelines [23], specifically (from 14 December 2022 to 21 March 2023).

### Study sample

The study sample comprised dental health professionals (Dentists, dental assistants, dental hygienists, and dental technicians) working in private and government healthcare sectors of Saudi Arabia. However, non-dental health professionals, dental students and interns were excluded from the study.

### Sample size and technique

The determination of the sample size was initially conducted using a formula for a single population proportion. This decision was based on the assumption that the % of dental healthcare professionals (DHPs) in Saudi Arabia possessed knowledge about impression disinfection during the COVID-19 pandemic was 50%. This assumption was made due to the absence of any reported studies on this topic in Saudi Arabia. The sample size 423 was determined by considering a 5% margin of error and a potential % non-response rate of 10%. The sample size was calculated based on the following formula:

$$ \varvec{n}=\frac{{\varvec{z}}_{\varvec{\alpha }}^{2} \varvec{p}\left(1-\varvec{p}\right)}{{\varvec{d}}^{2}}$$

Where n = is the desired calculated sample size, *Z*α = Standard normal variable at 95% confidence level (1.96); *p* = Proportion of knowing impression disinfection (50%); Confidence interval = 95%; Level of significance = 5%; *d* (margin of error) = 5% The sample size estimation yielded 423 subjects to be part of the study. Using an online data collection method, 800 participants responded within the specified timeframe and provide a rationale for the inclusion of these additional participants beyond the calculated sample size. Upon careful examination of the eligibility criteria and the subsequent exclusion of incomplete data, 718 DHPs were deemed suitable for inclusion in the final analysis.

The study participants were recruited utilizing snowball sampling techniques, which involved using the author’s network and popular social media platforms like Facebook, Telegram, and email. Additionally, officials of the Saudi dental society were requested to share the questionnaire link with the active registered DHPs. However, it must be acknowledged that the snowball sampling has some limitations such as risk of selection bias, limited representativeness, and increased dependence on current social networks of DHPs for obtaining the information.

### Questionnaire

A structured, close-ended, and self-administered questionnaire was prepared in English language based on ADA/CDC/MOH guidelines and previously published research on impression disinfection protocols [4, 24, 25].

#### i.Content of questionnaire

The questionnaire consisted of four parts: the first part comprised nine items on demographic information (gender, age, nationality, specialty, degree, years of experience, employment sector, and COVID-19 infection status) and the source of information on impression disinfection during COVID-19. The second part elicited the knowledge of impression disinfection through 18 items. The third part assessed respondents’ attitudes toward dental impression disinfection protocol during the COVID-19 pandemic. The last part assessed the respondent’s practice on impression disinfection.

The knowledge inquiries were accompanied by three possible responses for the seven items: true, false, or do not know. However, the question about the preferred impression technique for suspected COVID-19 patients deviated from this pattern, as it presented either the conventional method or the digital process. Similarly, participants were asked to answer five knowledge questions about impression disinfection methods. The options for disinfection methods included spraying, immersion, and using cotton soaked in disinfectant, and I do not know. The responses to materials used for disinfection comprised of phenols, iodophor, sodium hypochlorite, glutaraldehyde, and do not know.

The attitude of the study participants towards dental impression disinfection was evaluated through five questions with responses on five points Likert scale agreement (strongly agree, agree, neutral, disagree, and strongly disagree). Similarly, study participants’ practice towards impression disinfection was assessed through five items with four-point Likert scale responses (not sure, never, sometimes, and always).

#### ii.Validity and reliability

The questionnaire’s face validity was established by taking an expert opinion on every single question. The English version of the survey was forwarded to a dental public health specialist and an experienced prosthodontist to obtain their expert insights. The questionnaire was modified following the feedback provided by the experts. The reliability of the questionnaire was assessed by administering the questionnaire twice to 20 DHPs at an interval of one week. The reliability coefficient (Cronbach’s alfa) was 0.90, indicating an acceptable level of consistency.

#### iii.Questionnaire administration

An online English language questionnaire was prepared using Google Forms, and the link to the questionnaire was shared on the author’s contacts and popular DHP social media platforms in Saudi Arabia. The first section of the questionnaire provided participants with an overview of the study and clarified that their involvement was voluntary. The anonymity of the participants who completed the questionnaire was maintained. All the completed questionnaires in Google Forms were exported to Microsoft Excel 2016 for coding and then imported into special data analysis software programs. The questionnaires with missing one or more item responses in demographic or in knowledge, attitude and practice sections were excluded from the analysis. Moreover, questionnaires responded to by non-dental health professionals, dental students or interns were also excluded from the final analysis.

The data security and confidentiality were provided by the google form’s technical security features such as secure socket layer data encryption, creator access control, data storage in secure google server, two-factor authentication to sign in google account, audit logs and security patches. In addition, the chief investigator used strong, unique passwords, enabled two-factor authentication sign-in to the account, and avoided sharing the account permission to ensure data security and confidentiality utilizing online data collection methods.

#### iv.Statistical analysis of data

Before conducting the analysis, a value of 1 was assigned to all correct item responses, while inappropriate or incorrect responses were assigned a value of 0. The values were aggregated to generate a cumulative score for each participant. Furthermore, the individual scores for each Knowledge, Attitude, and Practice (KAP) domain were aggregated to derive a comprehensive overall score.

The Knowledge domain comprised of 18 questions dummy codes of “1” or “0” were assigned for the correct and incorrect responses, respectively. The total scores were calculated for each DHPs. Lastly, the class width is calculated by dividing the range (18–0 = 18) by 3 (desired number of the class interval) as shown in below equation:

Class width = Maximum Value − Minimum Value/Number of required class interval. Based on the derived class width (6), the total score was converted into three proportionate ordinal knowledge categories namely, ‘adequate’ (12.01–18), ‘average’ (6.01–12), and ‘inadequate’ (0–6). The adequate knowledge category indicates that the study participants were highly aware of the dental impression disinfection protocol during pandemic. Contrarily, average, and inadequate knowledge categories suggest moderate and poor awareness of dental impression disinfection. The participants with inadequate knowledge require extensive educational sessions, whereas average participants in knowledge categories may need less educational intervention. However, participants in adequate knowledge categories need no/least educational orientation towards impression disinfection protocol.

The domain of attitude, the categories were defined as ‘positive’ (scores ranging from 3.01-5), ‘neutral’ (scores ranging from 2.01 to 3), and ‘negative’ (scores ranging from 0 to 2). Similarly, the practice domain was categorized into adequate (3.1-5), average (2.1-3), and inadequate (0–2). Furthermore, the total KAP score was classified into three categories: ‘adequate’ (18.01–28), ‘average’ (9.01-18), and ‘inadequate’ (0–9).

A descriptive analysis was conducted to report the percentages and frequencies. The normality assessment of the data indicated a near-normal distribution of the scores. A multiple-response analysis was conducted to assess the source of information on the disinfection of impressions during COVID-19. The mean and standard deviation values were calculated for the knowledge, attitude, practice domains, and overall KAP items. An independent t-test was applied to compare the mean knowledge, attitude, and practice scores between genders, nationalities, employment status, and COVID-19 infection status.

Similarly, a one-way analysis of variance test (ANOVA) was applied to compare the mean scores among different age groups, specialties, qualifications, and years of experience. A Pearson’s test was applied to assess the correlation between the study participants’ knowledge, attitude, and practices towards impression disinfection protocol. All analysis was performed using IBM SPSS Statistics for Windows, version 25.0 (IBM Corp., Armonk, NY, USA). A value of *p* < 0.05 was considered significant for all the statistical tests.

## Results

### Sample characteristics

Of the 800 responses obtained, 718 DHPs completed questionnaires with a response rate of 89.75%. However, 82 (11.25%) questionnaires were removed from the analysis due to incomplete items and students’ responses. The sample mainly comprised male (53.6%) and middle-aged (31–40 years; 40.4%) DHPs. Most of the respondents were Saudi nationals (73.5%). Nearly 63.9% of respondents were dentists, followed by dental assistants (23.1%), dental technicians (7.5%), and dental hygienists (5.4%). Most participants had bachelor’s degree qualifications (50.8%) and 1–5 years of experience (35.8%). More than half of the participants were employed in the government sector (56.4%) and had a history of COVID-19 infection (50.3%), as shown in (Table 1).

**Table 1:** Demographic characteristics and COVID-19 status of the study participants (*N* = 718)

Variables		n	%	95% CL	
				Lower	Upper
Gender	Female	333	46.4	42.8	50.0
	Male	385	53.6	50.0	57.2
Age	20–30	289	40.3	36.7	43.9
	31–40	290	40.4	36.8	44.0
	> 41	139	19.4	16.6	22.4
Nationality	Non-Saudi	190	26.5	23.3	29.8
	Saudi	528	73.5	70.2	76.7
DHPs Specialty	Dentist	459	63.9	60.4	67.4
	DH	39	5.4	4.0	7.3
	DT	54	7.5	5.8	9.6
	DA	166	23.1	20.1	26.3
Qualification	Diploma	88	12.3	10.0	14.8
	Bachelor	365	50.8	47.2	54.5
	Postgraduate	242	33.7	30.3	37.2
	Others	23	3.2	2.1	4.7
Experience (in year)	< 1	81	11.3	9.1	13.8
	1–5	257	35.8	32.4	39.4
	6–10	179	24.9	21.9	28.2
	11–15	90	12.5	10.3	15.1
	> 15	111	15.5	13.0	18.2
Employment	Govt	405	56.4	52.8	60.0
	Pvt	313	43.6	40.0	47.2
COVID 19 Infection	No	357	49.7	46.1	53.4
	Yes	361	50.3	46.6	53.9

DH = Dental Hygienists, DA = Dental Technicians, DA = Dental Assistants, Govt = Government, Pvt = Private, CL = Confidence Level

**Fig. 1:**
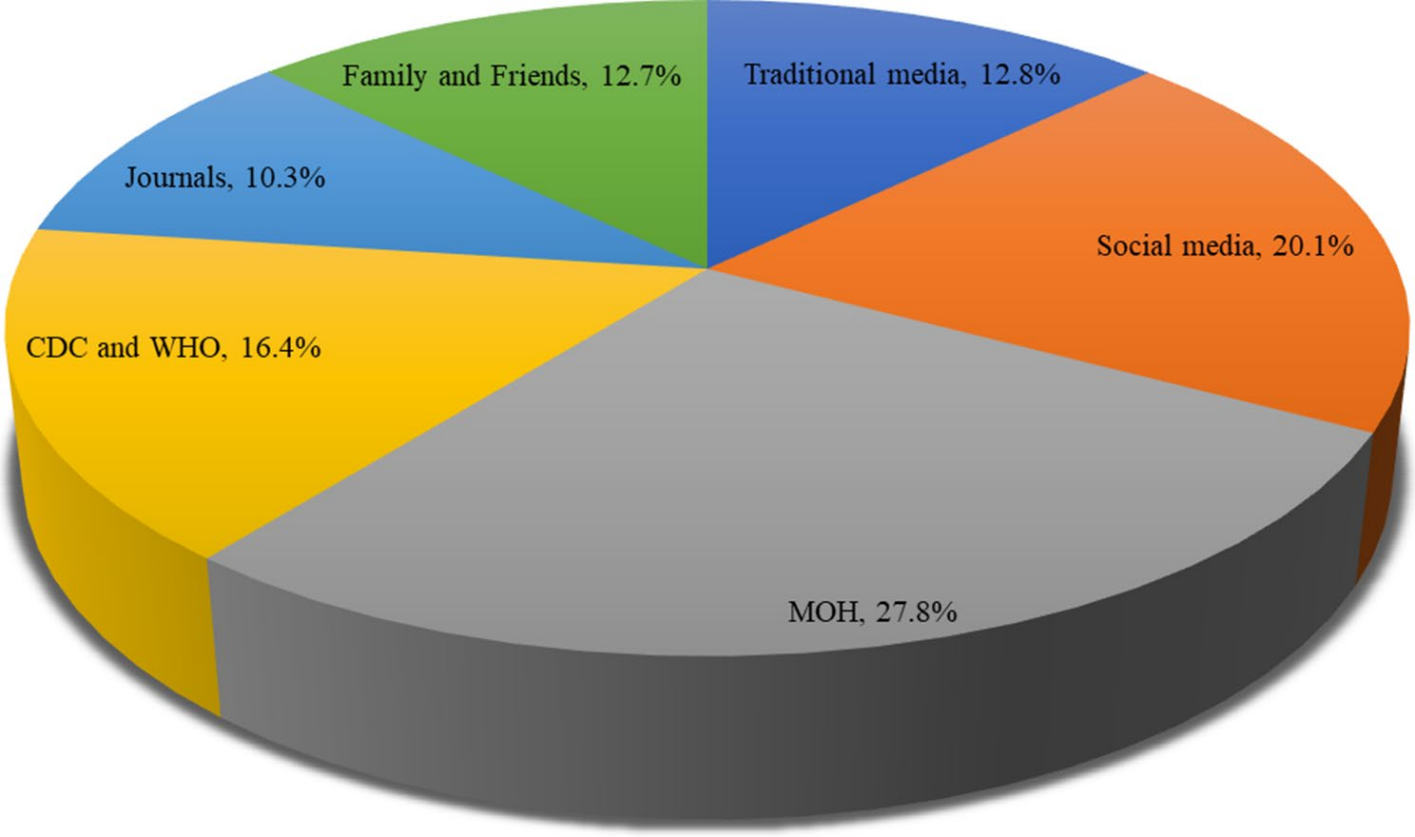
Source of information on disinfection of impression during COVID-19 (Multiple responses, *N* = 1559)

To evaluate the sources of information regarding disinfection of impressions, participants were asked to select multiple relevant options from the following: the MOH website, scientific journals, social media platforms, the CDC and WHO, traditional media, and family and friends. A dichotomized response (yes = 1 or no = 0) was collected for all the sources of information. A multiple response analysis was performed by inclusion of “yes” responses (*N* = 1559). The descriptive analysis of these responses showed that the MOH website was the most common source of information on disinfection of impression during the COVID-19 pandemic (27.8%), followed by the social media (20.1%), CDC and WHO (16.4%), traditional media (12.8%), family and friends (12.7%) and scientific journals (10.3%), as shown in (Fig. 1).

### Analysis of knowledge score

This study showed that most (54.6%) respondents had an average knowledge of impression disinfection. While 31.3% and 14.1% demonstrated adequate and inadequate levels of impression disinfection knowledge. Most respondents provided correct answers, except for the questions on the effective disinfection method utilized for disinfecting polysulphide, polyvinyl siloxane, polyether, and impression compound impression materials in which the spraying method was opted for by nearly half of the study participants. However, most of the study participants lacked the knowledge of suitable disinfectants used for various impression materials. However, study participants had relatively higher knowledge of taking digital impressions than conventional impressions during COVID-19. Poor knowledge of the impression disinfection was observed with the items; the COVID-19 pandemic has changed routine disinfection protocols for taking dental impressions (correct responses 22.4%), dental laboratories should have separate receiving areas for dental impression of suspected COVID-19 patients (correct responses 14.8%), disinfection method used for impression compound impressions of COVID-19 suspected patients (correct response 19.6%), chemical disinfectant used for disinfection of alginate impressions (correct responses 29.2%), and chemical disinfectant used for disinfection of polyether impressions (correct responses 16%) (Table 2).

**Table 2:** The knowledge of the impression disinfection among study participants (*N* = 708)

Knowledge items	Responses	n	%	95% CL	
				Lower	Upper
DHPs are at high risk of being infected with COVID-19 than general population	True	658	91.6%	89.5%	93.5%
	False	29	4.0%	2.8%	5.7%
	I dont know	31	4.3%	3.0%	6.0%
COVID 19 infection can spread through dental impression materials	True	542	75.5%	72.2%	78.5%
	False	83	11.6%	9.4%	14.1%
	I dont know	93	13.0%	10.6%	15.6%
COVID-19 pandemic has changed routine disinfection protocols of taking dental impression	True	381	53.1%	49.4%	56.7%
	False	161	22.4%	19.5%	25.6%
	I dont know	176	24.5%	21.5%	27.8%
Operatory attendant should carry the impression in container from dental operatory	True	527	73.4%	70.1%	76.5%
	False	51	7.1%	5.4%	9.2%
	I dont know	140	19.5%	16.7%	22.5%
While handling the dental impression of suspected COVID-19 patients use of universal protection	True	642	89.4%	87.0%	91.5%
	False	39	5.4%	4.0%	7.3%
	I dont know	37	5.2%	3.7%	7.0%
Dental laboratories should have separate receiving area for dental impression of suspected COVID-19 patients	True	533	74.2%	70.9%	77.3%
	False	106	14.8%	12.3%	17.5%
	I dont know	79	11.0%	8.9%	13.4%
DHPs should be aware about the disinfection procedure of impression received from suspected COVID-19 patient	True	646	90.0%	87.6%	92.0%
	False	25	3.5%	2.3%	5.0%
	I dont know	47	6.5%	4.9%	8.5%
Preferred impression technique	Conventional	225	31.3%	28.0%	34.8%
	Digital	493	68.7%	65.2%	72.0%
Disinfection methods of various impressions of COVID-19 suspected patients?	**Spraying** **%**	**Immersion** **%**	**Cotton soaked in disinfectant.** **%**	**Don’t know** **%**	
Alginate impressions	66.2	13.4	6.8	13.6	
Polysulphide impressions	51.1	21.4	4.5	23.0	
Polyvinyl siloxane impressions	49.7	23.3	5.6	21.4	
Polyether impressions	52.9	16.0	6.3	24.8	
Impression compound impressions	52.4	19.6	5.2	22.8	
Preferred disinfectants for various impressions in COVID-19 suspected patients?	**Phenols** **%**	**Iodophor** **%**	**Sodium Hypochlorite** **%**	**Glutaraldehyde** **%**	**Dont know** **%**
Alginate impressions	17.4	9.2	20.1	16.6	36.8
Polysulphide impressions	14.5	9.3	14.6	17.7	43.9
Polyvinyl siloxane impressions	12.7	8.8	16.0	19.5	43.0
Polyether impressions	13.1	9.5	15.6	16.0	45.8
Impression compound impressions	13.4	7.7	16.7	14.9	47.4

Knowledge score (Mean ± SD) 10.31 ± 3.22; Range (0–18); Inadequate 101 (14.1%), Average 392 (54.6%), Adequate 225 (31.3%); **CL = Confidence Level**

When the mean knowledge score of impression disinfection was compared between non-saudi (10.79 ± 3.01) and Saudi DHPs (10.14 ± 3.28), a statistically significant difference was observed (*p* = 0.013). Dental hygienists (7.49 ± 3.04) demonstrated a significantly lower knowledge than dentists (10.54 ± 3.23), dental assistants (10.55 ± 3.02), and dental technicians (9.69 ± 2.94) (*p* < 0.001). DHPs with postgraduate (10.64 ± 3.24) qualifications showed a significantly higher mean knowledge score than the DHPs with bachelor’s (10.31 ± 3.16), diploma (9.68 ± 3.33), and other (9.30 ± 3.30) qualifications. Similarly, DHPs with < 1 year of experience (11.11 ± 2.87) demonstrated a significantly higher mean knowledge score than those with 6–10 years of experience (9.93 ± 3.33). The knowledge domain score was 10.31 ± 3.22 (Table 3).

**Table 3:** Attitude and practice toward impression disinfection (*N* = 718)

Attitude toward impression disinfection	Strongly agree			Agree			Neutral			Disagree			Strongly disagree		
	%	95% CL		%	95% CL		%	95% CL		%	95% CL		%	95% CL	
		L	U		L	U		L	U		L	U		L	U
Dental Impressions of COVID-19 suspected patients should be rinsed thoroughly under running tap water before disinfection to remove as much bioburden as possible.	49.0	45.4	52.7	31.5	28.2	34.9	11.6	9.4	14.1	4.2	2.9	5.8	3.8	2.5	5.3
Washing hands is important before making impressions of COVID-19 suspected patients	59.5	55.8	63.0	24.0	20.9	27.2	12.3	10.0	14.8	2.2	1.3	3.5	2.1	1.2	3.3
Washing hands is important after making impressions of COVID-19 suspected patients	76.7	73.5	79.7	14.9	12.4	17.6	5.4	4.0	7.3	1.4	0.7	2.5	1.5	0.8	2.6
Washing tray is important before making of impressions of COVID-19 suspected patients	38.3	34.8	41.9	20.3	17.5	23.4	24.2	21.2	27.5	11.8	9.6	14.4	5.3	3.8	7.1
Scrubbing of the impression before disinfection is important in COVID-19 suspected patients	27.4	24.3	30.8	18.7	15.9	21.6	24.1	21.1	27.3	18.9	16.2	21.9	10.9	8.7	13.3
The practice of impression disinfection	**Always** **%**			**Sometimes** **%**			**Not sure** **%**			**Never** **%**					
Wash the impression of COVID-19 suspected patients immediately after removal	70.5	67.1	73.7	8.6	6.7	10.9	15.9	13.3	18.7	5.0		3.6		6.8	
Carryout disinfection of the impression of COVID-19 wearing personal protective equipment	73.8	70.5	76.9	11.7	9.5	14.2	9.7	7.7	12.1	4.7		3.4		6.5	
Follow disinfection protocol and store the disinfected impression of COVID-19 patients in a sealed plastic bag?	76.3	73.1	79.3	11.0	8.9	13.4	9.6	7.6	11.9	3.1		2.0		4.5	
Prefer digitalization of impressions of COVID-19 patients to reduce the risk of cross-contamination	55.0	51.4	58.6	20.6	17.8	23.7	17.5	14.9	20.5	6.8		5.2		8.8	
Indicate the risk of infection on the prescription and specify the disinfection practices carried out	51.8	48.2	55.5	24.9	21.9	28.2	17.1	14.5	20.0	6.1		4.5		8.1	

Attitude score (Mean ± SD) 3.03 ± 0.99; Negative 145 (20.2%), Neutral 393 (54.7%), Positive 180 (25.1%)

Practice score (Mean ± SD) 4.04 ± 1.39; Inadequate 100(13.9%), Average 69(9.6%),adequate 549 (76.5%) Score range (0–5)

### Analysis of attitude scores

One quarter (25.1%) of the participants showed a positive attitude, while more than half (54.7%) were neutral, and 20.2% demonstrated a negative attitude towards impression disinfection. The correct responses to disinfection protocol questions ranged from 17.1 to 91.6%. Washing hands after making impressions of COVID-19 suspected patients was the best attitude demonstrated by the study participants, followed by washing hands before making an impression (83.4%). Contrarily, participants showed poor attitude responses to washing tray is important before making impressions of COVID-19 suspected patients (correct response 17.1%) and scrubbing of the impression before disinfection is important in COVID-19 suspected patients (29.8%) (Table 4).

**Table 4:** Comparison of impression disinfection knowledge, attitude, and practice scores in different groups

Variables		Knowledge score			Attitude score			Practice score			Overall KAP		
		Mean	SD	p	Mean	SD	p	Mean	SD	p	Mean	SD	p
Gender	Female	10.23	3.29	0.510	3.06	0.96	0.342	4.09	1.40	0.432	17.38	4.38	0.984
	Male	10.38	3.17		2.99	1.02		4.01	1.39		17.38	4.14	
Age	20–30	10.37	3.24	0.409	2.87	1.05^A^	0.002	3.88	1.52	0.010	17.12	4.37	0.314
	31–40	10.13	3.19		3.09	0.96^B^		4.23	1.25		17.46	4.16	
	> 41	10.56	3.27		3.20	0.90^B^		4.00	1.37		17.76	4.18	
Nationality	Non-Saudi	10.79	3.01	0.013	3.10	0.98	0.226	4.24	1.21	0.013	18.13	4.01	0.003
	Saudi	10.14	3.28		3.00	1.00		3.97	1.45		17.11	4.31	
DHP Specialty	Dentist	10.54	3.23 ^A^	< 0.001	3.01	1.06	0.088	4.03	1.43^AB^	0.019	17.58	4.34^A^	< 0.001
	DH	7.49	3.04^B^		2.92	0.84		3.44	1.54 ^B^		13.85	3.73^B^	
	DT	9.69	2.94 ^A^		2.80	1.00		4.22	1.27 ^A^		16.70	3.98^A^	
	DA	10.55	3.02^A^		3.16	0.80		4.17	1.26 ^A^		17.89	3.80^A^	
Qualification	Diploma	9.68	3.33	0.045	2.90	0.87	0.015	4.09	1.39 ^AB^	0.044	16.67	4.45 ^BA^	0.007
	Bachelor	10.31	3.16		2.96	0.95		3.98	1.42 ^AB^		17.25	4.07 ^BA^	
	Postgraduate	10.64	3.24		3.19	1.01		4.18	1.30 ^A^		18.00	4.23^A^	
	Others	9.30	3.30		2.87	1.58		3.39	1.67 ^B^		15.57	5.53^B^	
Experience (in years)	< 1	11.11	2.87^A^	0.028	2.95	0.74 ^AB^	0.024	3.63	1.65^B^	0.041	17.69	3.72	0.219
	1–5	10.28	3.18 ^AB^		2.94	1.12^B^		4.04	1.36 ^AB^		17.25	4.35	
	6–10	9.93	3.33 ^B^		3.01	0.93 ^AB^		4.09	1.37 ^AB^		17.02	4.24	
	11–15	9.92	3.40 ^AB^		3.02	1.02^AB^		4.27	1.29^A^		17.21	4.63	
	> 15	10.74	3.15 ^AB^		3.31	.89^A^		4.11	1.34 ^AB^		18.15	4.05	
Employment	Govt	10.19	3.33	0.265	3.05	0.98	0.370	4.04	1.39	0.978	17.29	4.39	0.531
	Pvt	10.46	3.08		2.99	1.02		4.04	1.40		17.49	4.07	
COVID 19 Infection	No	10.25	3.31	0.629	3.01	1.04	0.602	3.90	1.54	0.006	17.16	4.50	0.164
	Yes	10.37	3.15		3.04	0.95		4.19	1.21		17.60	3.98	
Domain score (mean ± SD)		10.31 ± 3.22			3.02 ± 0.99			4.04 ± 1.39			17.37 ± 4.25		

Overall KAP: Inadequate 30 (4.2%), Average 373 (51.9%), Adequate 315 (43.9%)

The mean attitude score ranged from 0 to 5. DHPs showed an overall attitude score of (3.02 ± 0.99). DHPs in the age group of 20–30 years (2.87 ± 1.05) showed a significantly lower attitude score than those in 31–40 years (3.09 ± 0.96) and > 41 years (3.20 ± 0.90) (*p* = 0.002). Similarly, DHPs with postgraduate qualifications (3.19 ± 1.01) showed significantly higher attitude scores than those with bachelor (2.96 ± 0.95), diploma (2.90 ± 0.87), and other (2.87 ± 1.58) qualifications (*p* = 0.015) (Table 3).

### Analysis of practice scores

Adequate standards of practice were reported by more than three-fourths of the study participants (76.5%) of respondents, with only 13.9% having inadequate standards. Practice aspects related to awareness of disinfection of the impression wearing personal protective equipment (85.5%) and following disinfection protocol and storage of impression of COVID-19 suspected patients (87.5%) showed high standards (Table 4).

When practice score was compared across different age groups (*p* = 0.010), nationality (*p* = 0.013), specialty (*p* = 0.019), qualification (*p* = 0.041), and previous COVID-19 infection (*p* = 0.006), a statistically significant difference was observed. Participants aged 31–40 demonstrated higher mean practice scores than the other two age groups. Non-Saudi DHPs (4.24 ± 1.21) had significantly better practice towards impression disinfection than Saudi DHPs (3.97 ± 1.45). Dental hygienists (3.44 ± 1.54) demonstrated significantly poorer practice towards dental impression disinfection than dental technicians (4.22 ± 1.27) and dental assistants (4.17 ± 1.26). However, dentists’ practice scores did not differ significantly from other DHPs.

The DHPs with postgraduate qualifications (4.18 ± 1.30) showed better practice scores compared to those with diplomas (4.09 ± 1.39), bachelor’s (3.98 ± 1.42), and others (3.39 ± 1.67). However, postgraduate-qualified DHPs showed significantly higher scores than the DHPs with other qualifications. Similarly, a higher experienced DHPs 11–15 years (4.27 ± 1.29) and > 15 years (4.11 ± 1.34) showed better practice towards impression disinfection than less experienced DHPs < 1 year (3.63 ± 1.65) and 1–5 years (4.04 ± 1.36).

Similarly, DHPs who had COVID-19 infection (4.19 ± 1.21) showed a significantly higher impression of disinfection knowledge than those who did not have the19 infection (3.90 ± 1.54) (Table 3).

### Correlation between KAP

Pearson’s correlation revealed significant positive linear correlations between knowledge-attitude (*r* = 0.258, *p* < 0.01), knowledge-practice (*r* = 0.283, *p* < 0.01), and attitude-practice (*r* = 0.196, *p* < 0.01). This result supports the relationship between knowledge, attitude, and practice of dental impression disinfection measures in dental practice (Table 5).

**Table 5:** The correlation between impression disinfection knowledge, attitude, and practices

		Knowledge score	Attitude score	Practice score
Knowledge	Correlation coefficient	1	0.258^**^	0.283^**^
	*p*		< 0.001	< 0.001
Attitude	Correlation coefficient	0.258^**^	1	0.196^**^
	*p*	< 0.001		< 0.001
Practice	Correlation coefficient	0.283^**^	0.196^**^	1
	*P*	< 0.001	< 0.001	

**. Correlation is significant at the 0.01 level (2-tailed)

## Discussion

### Comparison with existing literature

The sudden emergence of the SAR-CoV-2 pandemic has increased the need to prevent and control cross-infection risk among DHPs. National and international health agencies have recommended cross-infection control guidelines to mitigate COVID-19. Hence the present study was undertaken to evaluate the knowledge, attitude, and practice of DHPs toward dental impression disinfection protocol during the COVID-19 pandemic in Saudi Arabia. The current study findings indicate that despite the average knowledge and neutral attitude, most (76.5%) of the study participants adequately practiced impression disinfection.

As dental treatment necessitates close proximity to patients, DHPs are continuously exposed to pathogenic oral fluids, which can significantly spread SARS-COV-2 infection [26]. Hence DHPs in this study were highly knowledgeable about the fact that they are at higher risk of COVID-19 than the general population. This finding is in line with several previously reported studies in which a large proportion of the DHPs demonstrated an awareness of the risk of COVID-19 [27, 28].

Dental practitioners and individuals seeking dental care are susceptible to contracting COVID-19, particularly in prosthodontics. This heightened risk arises from the generation of bioaerosols during dental handpiece-assisted teeth preparation, as well as the proximity to oral fluids during impression-making [29]. Nearly three-fourths of the study participants in this study agreed with the fact that COVID-19 infection can spread through dental impression materials. This finding is supported by Stoeva et al. [30], in which 78.9% of dental professionals were aware of the spread of infection via dental impressions.

The use of universal precautions to mitigate the COVID-19 spread among DHPs is well-documented in several studies [31, 32]. In this study, a large percentage of subjects were aware of applying universal protection in handling the dental impression. Moreover, most of the study participants were aware of carrying the dental impression in a container from the dental laboratory and allocating a separate area for receiving the dental impression of suspected COVID-19 patients. This finding aligns with a previous study in which more than half of the DHPs revealed that they have separate areas for receiving dental impressions in their laboratory [33].

Implementing cross-infection control measures plays a vital role in ensuring patient safety. Impression disinfection is a crucial measure that can effectively mitigate the transmission of infections between dental clinics, laboratory technicians, patients, and dental auxiliaries. The dentist is responsible for selecting an appropriate disinfection method for various impression materials [34]. In this study, 90% of the participants knew of the impression disinfection procedure. In contrast, only 21.9% of the dental technicians were aware of and employed infection control measures within the dental laboratory [33]. It could reflect the deeper concerns of the cross-infection threat posed by COVID-19 and periodic mitigation guidelines issued by the Ministry of Health to contain the SARS-CoV-2.

Various methods of disinfection are utilized to disinfect distinct types of impression materials. The chemical method is the primary approach among the different available ways. In this technique, a chemical disinfectant is administered to the surface of the impression materials. The disinfection mechanism of chemical agents may include protein coagulation, disruption of the cell membrane, removal of the free sulphydryl groups, and substrate competition [5].

The disinfection of impression materials can be accomplished through immersion or spraying various disinfectants at varying concentrations and durations. Spraying disinfectant is the recommended disinfection method for alginate and polyether impression materials, as prolonged immersion can cause distortion. Contrarily, silicone impression materials can be disinfected by immersion methods [25]. In this study, most participants relied on spraying disinfectant to disinfect COVID-19-infected impression materials. This finding is contrary to the previous studies in which the immersion disinfection technique was preferred over the spraying method of disinfection due to the complete coverage of the material by the disinfectant [33, 35]. In addition, more than 20% of the participants in our study did not know about the method of disinfection of polysulphide, polyvinyl siloxane, polyether, and impression compounds, suggesting a lack of disinfection knowledge on different impression materials.

Various disinfectants can be used for the disinfection of impression materials, including glutaraldehyde at concentrations of 0.5%, 2%, 2.2%, and 2.45%, sodium hypochlorite (NaOCl) at concentrations of 0.5%, 0.525%, 1%, 4%, and 5.25%, chlorine compounds at a concentration of 0.2% chlorhexidine, hydrogen peroxide at a concentration of 0.5%, iodophors at concentrations of 5% and 10%, and phenol compounds at a concentration of 7%. The choice of disinfectant depends on the type of impression material used [25]. In this study, most participants were unaware of the various chemical disinfectants used to disinfect the different impression materials.

The conventional method of making dental impressions involves the presence of a patient’s biological fluids, such as saliva or blood, which can contaminate SARS-CoV-2 among professionals involved in this process. The virus can persist in a humid environment until the impression reaches the prosthesis laboratory. If this impression is not properly disinfected, it can serve as a medium for transmitting the virus to the plaster models and subsequently to the professionals working in the prosthesis laboratory [36]. One potential strategy for reducing the transmission of viruses is implementing digital impression technology alongside a comprehensive workflow. The scanned image is transmitted to the prosthetic part manufacturing system through Computer-aided design/Computer-aided manufacturing (CAD/CAM) in the prosthesis laboratory. This measure effectively mitigates the transmission of contaminants within the chain of individuals involved in the delivery process to laboratories and among the personnel working in the prosthesis laboratory [36]. In this study, most of the study participants preferred using digital impression methods to make the impression of COVID-19 suspected patients.

It is widely recognized that various impression materials exhibit varying reactions to distinct disinfection methods, durations of disinfection, types of disinfectants, and concentrations thereof. Hence, it is imperative to adhere to the manufacturer’s impression materials guidelines to ascertain the appropriate disinfection approach [25]. Stoeva et al., 2024 found that 71.5% of study participant were aware of disinfectant protocols used for the impression materials [37]. In this study 31.3% of the participants had adequate knowledge of impression disinfection.

In our study sodium hypochlorite solution was the preferred agent for disinfection of alginate (20.1%) and impression compound impressions (16.7%). While gluteraldehyde was preferred choice for disinfecting the polysulphide (17.7%), polyvinyl siloxane (19.5%) and polyether (16%) impressions of COVID-19 suspected pateints. This study finding was supported by Stoeva et al. (2024), wherein, 31.9% and 28.5% knew about sodium hypochlorite and glutaraldehyde for disinfecting reversible and irreversible hydrocolloid impression materials [37].

In our study most of the participants agreed that spraying method of disinfection should be preferred while disinfecting the various impression materials obtained from the suspected COVID-19 patients. However, Stoeva et al. reported a high knowledge on use of Hermetic bag and soaked napkin, and dip technique to disinfect the irreversible and reversible hydrocolloid impression materials [37]. Moreover, in this study the impression disinfection knowledge was relatively higher in non-Saudi nationals, dentists, and dental assistants having postgraduate degree qualification and those having less than one year of experience.

The disparities in knowledge among the research participants in different studies may be attributed to the time of data collection, which occurred after the pandemic in Stoeva et al.‘s study. It is possible that the participants in their study acquired more information about impression disinfection compared to the DHPs in our study.

It has been recommended to rinse impressions using a continuous flow of water and/or gently scrub them with a camel hairbrush and a liquid detergent while under a continuous flow of water. This process aids in the elimination of bioburden. Applying dental stone sprinkled onto the impression and subsequent gentle scrubbing can effectively eliminate resistant substances [38]. The current study revealed that more than 80% of the study participants showed a positive attitude towards rinsing the COVID-19-infected dental impression thoroughly under running tap water, and less than half scrubbed the impression before disinfection to remove as much bioburden as possible. In accordance with our findings, prior studies revealed that 37.2–100% of the DHPs chose rinsing the impressions with tap water, despite the fact that rinsing alone did not totally eradicate contaminants [39, 40].

The World Health Organization (WHO) recommends using soap and water or alcohol-based sanitizers to mitigate the transmission of SARS-CoV-2 [41]. Given this, most of the DHPs in our study showed a positive attitude towards washing hands before and after taking the impression of the COVID-19 suspected patients. Moreover, nearly half of the participants agreed to wash the impression trays before taking the impression. This finding corroborates with the previous study reported by Amin et al. (2014) in which almost all the participants acknowledged the significance of hand hygiene before and after impression-making and washed the impression trays before making the impressions [42]. It should be emphasized that the washing trays before the impression suggests an unfavorable attitude as washing the sterilized trays may get contaminated before making an impression [42].

In this study, more than half of the DHPs demonstrated a neutral attitude, and one quarter demonstrated a positive attitude towards impression disinfection. This attitude differed significantly based on the age, qualification, and years of experience of the DHPs. However, attitudes towards impression disinfection did not differ significantly among different DHPs despite the higher mean scores among dentists and dental assistants.

The optimal timing for cleaning and disinfecting the impression material is following its removal from the patient’s oral cavity while ensuring that blood, saliva, or other biological residues do not have the opportunity to desiccate [25]. In this study, most DHPs washed the impression of COVID-19-suspected patients immediately after removal. This finding is in accordance with the previous studies in which immediate rinsing of the dental impression is recommended [43–45].

Healthcare personnel implement various measures to mitigate the risks associated with the transmission of infectious agents to both patients and fellow healthcare staff. The personal protective equipment (PPE) utilized in dental clinics encompasses a range of items such as disposable caps, gowns, gloves, face masks, and adoption of protective eyewear. It is strongly advised to utilize them consistently during patient treatment and when managing patients’ impressions [46]. In line with this, our study showed that most DHPs wore personal protective equipment while carrying out impression disinfection. Moreover, the Ministry of Health guidelines in Saudi Arabia have strongly recommended using personal protective equipment while dealing with patients at risk of COVID-19 [17].

After using the spray or immersion method of disinfection of the dental impression, it is kept in a sealed plastic bag to utilize the manufacturer’s recommended time to increase the efficacy of the disinfecting agents [47, 48]. In this study, more than 87% of the DHPs followed the disinfection protocol and stored the disinfected impression of COVID-19 patients in a sealed plastic bag. This finding is significantly higher than the study reported by Muller-Bolla et al. in European Union Dental Schools, in which only 38% of cases stored the disinfected impression of patients in a sealed plastic bag [49].

The COVID-19 pandemic has accelerated the integration of digital technology in dentistry, positioning it as a vital and indispensable tool. This is primarily attributed to its capacity to ensure safety, enhance workflow efficiency, and potentially augment profitability [36, 50, 51]. In our study, more than three-fourths DHPs preferred digitalization of the impressions to reduce the risk of contamination during COVID-19. This finding is in with the previous study in which more than 85% of participants supported the adoption of digital dental technology protocols amidst the COVID-19 pandemic [52].

DHPs should affix labels onto impressions sent to dental laboratories, indicating the disinfection status of said impressions. Given the potential for alterations in dimensional stability and surface detail reproduction, DHPs must communicate effectively regarding the repetitive disinfection of impressions [53]. In this study, more than three-fourths of the DHPs indicated the risk of COVID-19 infection on the prescription and specified the disinfection practices.

In general, most of the DHPs demonstrated an adequate impression disinfection practice. However, adequate impression disinfection practices and a significant variation in mean practice scores were observed across age, nationality, specialty, qualification, experience, and past COVID-19 infection status of the DHPs. Dental hygienists showed a poor impression of disinfection practice than other DHPs groups. This could be because dental hygienists are mainly involved in scaling, polishing, and oral hygiene instruction rather than dealing with dental impressions. Therefore, this finding suggests a further need for improvement in impression disinfection practices.

The study findings further support the connection between knowledge, attitude, and practice regarding impression disinfection, as evidenced by the positive correlations between knowledge-attitude, knowledge-practice, and attitude practice. The present study establishes that having sufficient knowledge is associated with developing a positive attitude, which leads to adopting beneficial practices. The findings align with the results reported by previous studies [54, 55].

### Implications for practice

The results of this study reveal that a significant proportion of the DHPs exhibited an average level of knowledge, attitude, and practice toward impression disinfection. More than half of the study participants were in favour of spraying method for disinfecting dental impressions. However, most of the participants were also lacking information on the preferred disinfectant solution to be used for different types of impression materials. The current study findings highlighted lacunae in the knowledge, attitude, and practice of impression disinfection among DHPs during the COVID-19 pandemic. This information can be effectively utilized by the dental directorate and ministry of health in Saudi Arabia to develop guidelines on continuing educational programs and training of the DHPs on impression disinfection protocols to mitigate the spread of COVID-19 via dental impressions to improve the clinical practice in Saudi Arabia. Thus, enhancing the DHP’s knowledge, attitudes, and practices toward cross-infection control during pandemic and safeguarding the health of the DHPs and dental patients.

One notable aspect of this study is its comprehensive scope, encompassing a nationwide survey examining the knowledge, attitudes, and practices of DHPs regarding impression disinfection. It is worth highlighting that this survey was conducted during the COVID-19 pandemic, which adds relevance and timeliness to the findings. Adequate sample size consideration is another important aspect of the study.

### Limitations of the study

Unlike other studies this study also has some limitations, such as lower participation of dental hygienists and dental technicians than dentists and dental assistants might affected the results. It could be due to the fact that the dental technicians and dental hygienists constitutes lowest number of entire dental workforce in Saudi Arabia.

The study’s findings were based on self-reported data, which introduces the possibility of participant bias. The validity of the self-reported questionnaire is contingent upon cognitive factors and situational factors. Cognitive difficulties pertain to the respondents’ comprehension of the topic and their capacity to provide appropriate answers based on their knowledge and recall. Moreover, the survey was conducted during the COVID-19 pandemic leading to the socially desirable response from the study participants. Self-reported data are valid when participants comprehend the questions and when there is a sense of anonymity and slight apprehension of reprisal.

Although Arabic is the primary language of the study participants, English may have served as an impediment for those who were uncomfortable with it, influencing their responses. Moreover, technical reasons like website crashes or sluggish loading times may have affected the completion of the online survey by the participants. Additionally, the study only included individuals actively engaged on social media, potentially leading to a selection bias that could impact the generalizability of the results.

Typically, a cross-sectional study is limited to identifying an association between risk factors and outcomes without the ability to establish a causal relationship.

### Future research

It is recommended that future follow-up studies be conducted using a nationwide representative sample of DHPs, including dentists, dental hygienists, dental technicians, and dental assistants. These studies should aim to assess the DHPs’ knowledge and practices of the preferred disinfectants and methods for disinfecting impressions made with different materials commonly used in dental practice. Furthermore, future research questionnaires should incorporate the items on utilization of microwaves and Ultraviolet light in impression disinfection.

### Causal relationships

This is a cross-sectional study and, therefore, cannot establish causal relationships.

## Conclusion

DHPs who participated in this study exhibited average knowledge and attitudes, along with adequate adherence to proper practices regarding impression disinfection amidst the COVID-19 pandemic. The existing understanding of the chemicals and techniques employed in disinfecting dental impressions is lacking and necessitates enhancement by conducting regular and ongoing dental educational programs on proper disinfection techniques of dental impressions.

## Data Availability

The datasets used and analyzed during the current study are available from the corresponding author on reasonable request.

## Declarations

The ethics committee of research and innovation center of Riyadh Elm University formally approved the study after careful review of the research proposal (IRB number: FPGRP/2022/701/807/776). This study was carried out in conformity with the Helsinki Declaration involving human participants. The DHPs participation in the study was voluntary. The purpose of the study, the methods of data acquisition, and the potential risk and benefits were explained in detail to the DHPs. An informed consent to participate in the study was obtained from all the DHPs.

## Abbreviations

DHPs: Dental Health Professionals
DA/: Dental Assistant
DT: Dental laboratory technician
DH: Dental Hygienist
KAP: Knowledge Attitude and Practice
